# Editorial: Effects of vascular function and aging on brain circulation and neurodegeneration

**DOI:** 10.3389/fragi.2024.1385066

**Published:** 2024-03-05

**Authors:** Benjamin Petersen, Sharon Negri, Madison Milan, Helen Shi, Zeke Reyff, Cade Ballard, Jennifer Ihuoma, Andrea Di Francesco, Stefano Tarantini

**Affiliations:** ^1^ Oklahoma Center for Geroscience and Healthy Brain Aging, University of Oklahoma Health Sciences Center, Oklahoma City, OK, United States; ^2^ Vascular Cognitive Impairment and Neurodegeneration Program, Department of Neurosurgery, University of Oklahoma Health Sciences Center, Oklahoma City, OK, United States; ^3^ Calico Life Sciences LLC, South San Francisco, CA, United States; ^4^ Peggy and Charles Stephenson Cancer Center, University of Oklahoma Health Sciences Center, Oklahoma City, OK, United States; ^5^ Hudson College of Public Health, University of Oklahoma Health Sciences Center, Oklahoma City, OK, United States; ^6^ International Training Program in Geroscience, Doctoral School of Basic and Translational Medicine/Department of Public Health, Semmelweis University, Budapest, Hungary

**Keywords:** dementia, cognitive function, blood-brain barrer, neurovascular function, vascular oxidative stress

The aging process prompts morphological, structural, and functional changes in the vasculature of our bodies (Weijs et al., 2023). These alterations occur in both health and disease and serve as mechanistic foundations for many cardiovascular and cerebrovascular diseases that are prevalent in the aging population (Vestergaard et al., 2022; Lu et al., 2023). Cardiovascular and cerebrovascular diseases are the most common cause of death among older people in the United States (Jaul and Barron, 2017), accounting for nearly 1/3rd of all deaths in the United States by the age of 65, and approximately 2/3rd of all deaths by the age of 85. With the percentage of the population over the age of 65 set to increase from 12% to 22% in the next 30 years (Ferruc et al., 2008), understanding and treating age-related vascular disease is paramount (Bloom et al., 2022).

Aging in the brain is characterized by a vast array of functional and structural alterations of the microcirculation (Schulz et al., 2022; Negri et al., 2023), contributing to the pathogenesis of age-related diseases including vascular cognitive impairment, Alzheimer’s disease, and mild cognitive impairment (Williamson et al., 2024). Growing interest and understanding of the role of the aging vasculature in the context of the age-related loss of cognitive function (Gorelick et al., 2011) has prompted many investigative efforts to better understand the macro and microvascular mechanisms underlying the development of dementia (Yabluchanskiy et al., 2021).

The collection of papers published in the Research Topic: *Effects of vascular function and aging on brain circulation and neurodegeneration* underlines the growing interest and understanding of the role of the aging vasculature, in the context of age-related neurodegeneration. The goal of this Research Topic is to gather and summarize the evidence that relates to the mechanisms underlying the neurodegenerative disease of aging.

In an original article, Zhang et al. retrospectively analyzed the neurological outcomes of 408 patients with acute anterior circulation strokes treated with mechanical thrombectomy who were concomitantly in hyperglycemic states. The study stratified the patients into four quartiles based on the stress hyperglycemia ratio (SHR), defined as the fasting plasma glucose (mmol/L) divided by glycosylated hemoglobin (%). Interquartile comparison of functional neurologic outcomes, determined by the modified Rankin Scale, was performed at the 3-month follow-up. The findings of the study suggest that stress hyperglycemia, as indicated by higher SHR, is independently associated with increased risks of 3-month all-cause mortality and intracerebral hemorrhage, as well as lower rates of major neurological improvement following an anterior circulation stroke treated with mechanical thrombectomy. Importantly, these associations were observed irrespective of the diabetic status of the patient. The study suggests that the SHR can be a useful quantitative indicator of adverse outcomes and functional neurologic recovery in patients undergoing mechanical thrombectomy for acute anterior circulation stroke. Ultimately, this paper establishes an important framework in which future investigations can seek to determine ideal glucose control targets in the setting of an acute ischemic stroke treated with mechanical thrombectomy.

The association between the hemoglobin to red cell distribution width ratio (HRR) and clinical outcomes in acute ischemic stroke (AIS) patients undergoing mechanical thrombectomy (MT) was explored by Feng et al. in an original article. Researchers measured HRR at the time of admission and 24 h post mechanical thrombectomy. The study, consisting of 310 patients in total, revealed that those who died or had a poor prognosis following the stroke had significantly lower HRR levels at both time points. While HRR at admission was not linked to outcomes, HRR after 24 h was independently associated with poor prognosis and death. The study suggests that HRR, a rapidly measurable marker, could serve as an independent prognostic indicator for AIS patients undergoing mechanical thrombectomy, ultimately providing a valuable clinical tool.

Wang et al. reviewed the existing literature on the underlying mechanisms of myogenic, metabolic, and endothelial factors that contribute to cerebral blood flow autoregulation and the potential impact of impaired cerebral blood flow autoregulation on cognitive impairment. The review challenges the traditional understanding of Alzheimer’s Disease (AD) as a neurodegenerative disease solely related to beta-amyloid and tau proteins, as current treatments targeting these factors have been inadequate. The role of cerebrovascular dysfunction in AD development is explored, citing multiple studies demonstrating vascular dysfunction preceding Aβ accumulation and cognitive impairment. Additionally, the paper presents a new hypothesis of a three-line myogenic response defense mechanism for cerebral blood flow dysregulation. Wang et al. hypothesize that the loss of cerebral blood flow autoregulation may limit blood flow to ischemic regions, or conversely, increase blood flow and pressure to the vasculature of the brain, leading to decreased capillary and blood-brain barrier integrity and exacerbating cognitive impairment.

In a systematic review, Zhang et al. evaluated the association between intracranial and extracranial atherosclerotic stenosis and white matter hyperintensities, which are key neuroimaging manifestations of cerebral small vessel diseases. In this review and meta-analysis of twenty-one eligible studies, extracranial atherosclerotic stenosis was significantly associated with increased incidence and volume of white matter hyperintensities, whereas intracranial atherosclerotic stenosis was insignificantly associated with white matter hyperintensities. The study highlights the impact of white matter hyperintensities on various adverse outcomes, including cognitive impairment and stroke risk. The meta-analysis underscores the need for longitudinal studies to further evaluate the relationship between white matter hyperintensities and cognitive impairment as well as to assess the potential of interventions in preventing white matter hyperintensity progression.

Liu et al., in an original article, also investigated white matter hyperintensity progression in response to optimal systolic and diastolic blood pressure control. A longitudinal retrospective study was conducted, including 457 patients classified based on white matter hyperintensity severity. The findings from the study suggested that both baseline and longitudinal mean systolic blood pressure (SBP), diastolic blood pressure (DBP), and SBP standard deviation were significantly associated with white matter hyperintensity severity. An average SBP of 130–140 mmHg was associated with a higher risk of white matter hyperintensity progression, while a target means SBP of <130 mmHg and mean DBP of <80 mmHg were associated with a lower risk of white matter hyperintensity progression. The study suggests that controlling SBP and DBP within specific thresholds could be crucial in preventing or slowing white matter hyperintensity progression, providing insights into optimal blood pressure management for individuals at risk of cognitive dysfunction associated with white matter hyperintensities.

The evidence presented in these articles complements the existing body of work surrounding cerebrovascular aging by providing further insight into the intricate relationships between cerebrovascular structure, function, and age-associated cognitive decline. The aging process induces significant changes in the vasculature, contributing to the development of cerebrovascular diseases, which is a major cause of mortality among older individuals. While these studies provide valuable insights, it is crucial to acknowledge the limitations and advocate for further research to enhance understanding of these intricate relationships and pave the way for more effective preventive and therapeutic strategies in the realm of age-related neurodegenerative diseases.
